# A victim of its own progress: why the success of modern therapeutics forces a re-evaluation of placebo-controlled trials in migraine prevention

**DOI:** 10.3389/fneur.2026.1871028

**Published:** 2026-07-09

**Authors:** Nirit Lev, Leah Borovoi, Emily Elefant, Erel Domany, Oved Daniel, Gal Ifergane

**Affiliations:** 1 Department of Neurology, Meir Medical Center, Clalit Health Services, Kfar Saba, Israel; 2 Gray Faculty of Medical and Health Sciences, Sagol School of Neuroscience, Tel Aviv University, Tel Aviv-Yafo, Israel; 3 Azrieli Faculty of Medicine, Bar-Ilan University, Safed, Israel; 4 Department of Neurology, Rambam Health Care Campus, Haifa, Israel; 5 Neurology Division, Tel Aviv Sourasky University Medical Center (Ichilov), Affiliated to the Faculty of Medical and Health Sciences, Tel Aviv University, Tel Aviv-Yafo, Israel; 6 Department of Neurology and Clinical Research Center, Soroka University Medical Center, Faculty of Health Sciences, Ben Gurion University of the Negev, Be'er Sheva, Israel

**Keywords:** calcitonin gene-related peptide (CGRP), clinical trial design, declaration of Helsinki, migraine, neuroethics, placebo, placebo-controlled trials

## Introduction

Migraine is one of the most disabling neurological disorders worldwide and is a leading cause of years lived with disability (1, 2). The introduction of calcitonin gene-related peptide (CGRP)-directed monoclonal antibodies and gepants has moved prevention from a largely empirical, symptom-management paradigm toward mechanism-based therapy that can plausibly alter disease trajectory. That therapeutic shift raises a decisive neuroethical question: can it still be ethically acceptable to randomize participants with frequent migraine to months of placebo when highly effective, migraine-specific preventive options exist?

The methodology of clinical trials in neurology has long been dominated by the requirement for a placebo arm to establish absolute efficacy. In the late 20th century, the use of a placebo in migraine trials was a straightforward ethical decision. With few proven preventives, researchers needed a no-treatment baseline to identify a signal amidst the noise. In that era, the potential benefit of identifying any effective treatment outweighed the temporary discomfort of the placebo group. Placebo controls have long been defended in migraine because outcomes are variable and placebo response is substantial, and because active-control trials can lack assay sensitivity (3, 4).

However, clinical ethics is not static; it is a function of current medical capability and clinical equipoise. In the CGRP era, for many prevention-trial scenarios, prolonged placebo exposure is no longer a scientifically necessary compromise; it is an ethically avoidable harm. This perspective examines why the historical justifications for placebo-controlled trials are failing as the therapeutic landscape advances.

## The ethical framework: a changing calculus

The ethical governance of human experimentation is anchored by the Declaration of Helsinki (DoH) and the Council for International Organizations of Medical Sciences (CIOMS). These documents generally restrict placebo use to specific contexts: when no proven treatment is available, when the risk is negligible, or when compelling methodological reasons exist (5–7).

In modern migraine prophylaxis, the “no proven treatment” condition is no longer met. With the advent of CGRP-pathway inhibitors and established oral preventives (anticonvulsants, antidepressants, and antihypertensives), the “no treatment” scenario is an artificial construct of the trial design rather than a clinical reality. Furthermore, Article 33 of the DoH contains a critical caveat: placebos are only permissible if patients are not at risk of serious or irreversible harm.

### Standard-of-care shift in the CGRP era: consensus vs. access constraints

A key premise of ethical analysis is whether a proven-effective intervention exists that constitutes an acceptable standard of care. In 2024, the American Headache Society (AHS) updated its position statement, concluding that CGRP-targeting therapies should be considered a first-line approach for migraine prevention, without requiring prior failure of other preventive classes (8). This is a clinically and ethically significant shift: it explicitly places CGRP therapies alongside established first-line options, reframing them as mainstream prevention rather than exceptional rescue (9).

At the same time, the standard of care is not uniformly experienced by patients. In the United Kingdom, for example, the National Institute for Health and Care Excellence (NICE) technology appraisal guidance for atogepant recommends use only after failure of at least three preventive medicines (10). Such restrictions are not merely administrative: they structure who can access migraine-specific preventives and when. When effective treatments are available but differentially accessible, clinical trials risk becoming a de facto access pathway, which may compromise the voluntariness of participation and complicate the justice analysis. These access gradients also intensify international variation in the “best proven intervention” against which new agents should be evaluated (11, 12).

## When is placebo ethically permissible? Requirements of major guidance documents

Placebo controls are not categorically prohibited by research ethics. Both the Declaration of Helsinki and CIOMS accept a placebo in defined circumstances, especially where no proven effective intervention exists, or compelling methodological reasons require a placebo, and participants will not be exposed to an additional risk of serious or irreversible harm (5, 6). ICH E10 likewise recognizes placebo control as one option among several control-group choices, but stresses that ethical and scientific considerations must be balanced in selecting a control (13). The American Medical Association's Code of Medical Ethics similarly emphasizes that placebo controls require careful justification, that severe or painful conditions demand thorough exploration of alternatives, and that studies involving death or irreversible damage cannot ethically employ a placebo when an alternative therapy would prevent or slow progression (14, 15).

Across these frameworks, placebo is generally permissible only under four conditions: (i) no proven effective treatment exists; (ii) withholding treatment poses at most transient discomfort or delay in symptom relief; (iii) placebo is methodologically necessary to establish efficacy, without serious or irreversible harm; or (iv) participants would not otherwise have access to effective treatment and the research is responsive to the host setting (5, 6, 13, 14). Even when a placebo is permitted, safeguards are non-negotiable: informed consent that makes the rationale and implications of the placebo explicit; minimization of placebo duration; rescue and early-escape criteria; robust safety monitoring and interim analyses; and, where appropriate, post-trial access to effective therapy (5, 6, 16).

The ethical analysis of placebo use in migraine research has frequently drawn on arguments developed in the context of acute-treatment trials, where placebo exposure is brief, rescue medication is available within hours of a single attack, and the harm of a failed trial visit is largely confined to one episode of pain and functional disruption (17). This ethical reasoning cannot be applied to migraine preventive trials, which must occupy a fundamentally different ethical territory. Conflating the ethical justification of acute- and preventive-treatment trials understates the burden borne by participants assigned to placebo in prevention studies.

In acute trials, the unit of placebo exposure is a single migraine attack. A participant who receives a placebo for one attack experiences that attack fully but is then free to resume any preventive or acute therapy they were previously using. Rescue medication is typically protocol-specified and available within the same episode, often within 2 h of treatment failure. The ethical cost of a placebo in this context is real but bounded: it is the difference between a treated and an untreated attack, measured in hours.

In preventive trials, the unit of placebo exposure is a season of neurological disease. Standard parallel-group designs expose participants to 12 to 24 weeks of placebo, during which they experience the full, unattenuated burden of their underlying migraine frequency. The ethical risks derived from such prolonged exposure, e.g., disease progression toward chronification, development of medication-overuse headache, and cumulative functional disability, have been discussed above. The ethical asymmetry with acute trials is sharp: in acute trials, the availability of rescue medication within the episode is a safeguard; in preventive trials, increased reliance on acute medication across the placebo period is the hazard. What functions as protection in one trial design becomes the mechanism of harm in the other.

These convergent standards sharpen the ethical question in migraine prevention in the CGRP era: first, does a proven effective preventive treatment exist? And, second, would extended placebo exposure impose more than negligible, reversible risk? Table 1 maps these criteria to contemporary migraine prevention trials. Broader neuroethical analyses of placebo emphasize the distinct ethical risks of placebo use in fluctuating neurological conditions, where symptom recurrence is itself disabling (4, 15, 18).

**Table 1:** Ethical permissibility of placebo controls: core conditions and implications for migraine prevention trials.

Ethical condition (Helsinki/CIOMS/ICH)	Standard requirement	Relevance to migraine prevention in the CGRP era	Design implication
No proven effective intervention exists (5, 6, 13)	Placebo is acceptable when no established, effective therapy is available to withhold	Not met in most current preventive-trial contexts. CGRP-targeting therapies are established as first-line prevention (8, 9), rendering the “no proven treatment” justification inapplicable for the majority of prevention-trial scenarios	Use an active comparator. Placebo may be considered only in narrowly defined subpopulations for whom no proven option exists or to whom existing evidence cannot be extrapolated
Withholding treatment poses at most temporary discomfort or delay in symptom relief (6, 13)	Delaying active therapy must not expose participants to clinically significant morbidity	Frequently not met. Untreated migraine confers substantial disability (1, 2). Prolonged placebo exposure increases acute-medication use and the risk of medication-overuse headache and chronification (18, 19, 26). Disease-progression risk further strengthens the case for early, effective intervention (20)	If a placebo arm is retained, minimize its duration; mandate rescue medication and early-escape criteria; exclude patients with high baseline frequency, prior preventive failure, or risk factors for chronification
Compelling methodological reasons exist, without imposing additional risk of serious or irreversible harm (5, 6, 13)	A placebo may be necessary to establish assay sensitivity when outcomes are symptom-driven, fluctuate over time, and placebo response is substantial	Methodological concerns remain legitimate: placebo response in migraine prevention is large and heterogeneous (22, 23). However, the scientific rationale must be balanced against the foreseeable harms of prolonged placebo exposure (15, 25). Assay sensitivity can be preserved through design modifications rather than extended placebo arms	Adopt hybrid designs that preserve assay sensitivity while limiting placebo exposure: add-on designs, three-arm trials (active + placebo + experimental), crossover designs, or short placebo windows with pre-specified rescue criteria
Research is conducted in settings where effective interventions are unavailable, with host-population benefit and appropriate safeguards (6, 7)	Placebo may be permissible where participants would not otherwise access effective therapy, provided the research is responsive to local health needs and post-trial access is ensured	Directly relevant to the CGRP era. Payer restrictions and prior-authorisation requirements in multiple healthcare systems (10–12) mean that trial enrolment can function as a de facto access pathway. This structural vulnerability may compromise the voluntariness of consent and does not justify prolonged placebo exposure; it demands additional safeguards	Require enforceable post-trial access plans for participants who benefited or who remain in need of effective prevention. Do not rely on access inequities as justification for a placebo arm. Apply enhanced informed-consent procedures that explicitly address the access context

AHS, American headache society; CIOMS, council for international organizations of medical sciences; CGRP, calcitonin gene-related peptide; ICH, international council for harmonization; MOH, medication-overuse headache.

### Modern understanding: the risk of serious harm

The ethical defense of placebos often relies on the reductionist assumption that migraine causes only “temporary discomfort.” Modern neuroscience refutes this, highlighting two major concerns.

#### Neuroplasticity and disease chronification

Migraine is a dynamic disorder characterized by progressive neuroplastic changes. Frequent, uncontrolled attacks are associated with central sensitization, a state of heightened neuronal excitability in which the nervous system becomes increasingly reactive to both migraine-specific and non-specific triggers (19–23). Epidemiological and prospective observational data consistently identify high attack frequency, high acute medication use, and inadequate prophylaxis as the principal modifiable risk factors for the transition from episodic to chronic migraine (≥15 headache days per month) (24–26). Once established, chronic migraine is substantially more refractory to treatment, is associated with greater disability and psychiatric comorbidity, and represents a qualitatively worse disease state than the episodic form from which it arose.

Though direct evidence linking assignment to a placebo arm within a clinical trial to irreversible disease progression is limited, the available evidence chain is biologically plausible and ethically consequential: high-frequency uncontrolled attacks sustain the neurobiological conditions associated with chronification risk, and placebo assignment in a preventive trial plausibly sustains those conditions for the duration of the placebo period. The ethical argument does not require proof of causation; it requires foreseeability of harm. Critically, the risk is not binary but cumulative; the longer a participant remains in an uncontrolled high-frequency disease state, the greater the neuroplastic burden they accrue. A brief placebo run-in may entail limited and arguably acceptable risk; a 24-week placebo arm, by contrast, entails months of sustained exposure to conditions most strongly associated with disease progression (24–26). When effective prevention exists, cumulative exposure is not a scientific necessity. It is a foreseeable and preventable harm.

#### The burden of functional disability

According to the Global Burden of Disease studies, migraine is the leading cause of disability-adjusted life years (DALYs) in people under 50 (1, 2). In a modern context, withholding effective treatment for months is not a “minor delay”; it is a significant disruption of economic productivity, familial roles, and mental health. A patient assigned to a placebo is not merely “waiting”; they are losing months of their life to a preventable neurological event.

## Why placebo persists: assay sensitivity, bio-creep, and the challenge of non-inferiority

The historical reliance on placebo in migraine trials is not arbitrary, and a rigorous ethical argument must engage honestly with the methodological reasons it persists. Migraine outcomes are symptom-driven, fluctuate over time, and are highly susceptible to expectation effects. The principal defense for placebo-controlled trials is *assay sensitivity*, the ability of a trial to detect a true treatment effect when one exists, and thereby to avoid false-negative results that could either suppress effective therapies or permit approval of ineffective ones. The International Headache Society (IHS) and regulatory bodies such as the FDA have traditionally argued that, without a placebo, it may be impossible to determine whether a trial failed because the new drug was ineffective or because the trial itself was insufficiently sensitive to detect a difference. These concerns are not merely institutional conservatism; they reflect genuine methodological vulnerabilities that any proposed alternative to placebo-controlled designs must directly address.

## The problem of assay sensitivity in active-comparator designs

Placebo response rates in preventive migraine trials are substantial and heterogeneous. A meta-analysis of placebo response in migraine prophylaxis reported a pooled ≥50% responder rate of approximately 21%, with wide variation across studies and designs (27). A 2025 meta-analysis of placebo arms from pivotal phase 2/3 preventive trials found that placebo reductions in monthly migraine days differed significantly by region and patient characteristics, with higher placebo responses in North America and among participants without prior prophylactic use (28). Diener and colleagues argued that placebo controls remain important precisely because active-comparator trials can fail to distinguish the new drug from an active comparator that is itself underperforming in that particular trial population—a scenario where a genuinely ineffective new treatment could appear non-inferior (29). This risk is real: if the active comparator does not “work” in each trial setting due to regression to the mean, selection effects, or high background variability, any comparator, including an inert one, will appear to meet the non-inferiority threshold. The field cannot assume that an established drug will reliably reproduce its pivotal trial effect size in every subsequent trial population.

This concern, however, does not mandate a binary choice between placebo and active-comparator designs. Temple and Ellenberg argued that ethical and scientific considerations should be evaluated in concert, and that where active-control designs risk false-negative or uninterpretable results, hybrid designs can address both assay sensitivity and participant protection (30). Concretely, three-arm designs incorporating a new agent, an active comparator, and a time-limited or dose-capped placebo arm can preserve the internal validity that placebo provides while substantially reducing per-participant placebo exposure duration. Pre-specified interim analyses with early stopping rules can further curtail placebo arm duration once an efficacy signal is established.

## The problem of bio-creep in sequential non-inferiority trials

A second and underappreciated methodological risk is *bio-creep*, the gradual erosion of demonstrated efficacy across successive non-inferiority trials. If drug B is shown to be non-inferior to drug A, and drug C is subsequently shown to be non-inferior to drug B, the implicit assumption is that drug C retains some fraction of the efficacy originally demonstrated by drug A against placebo. But if the effect of drug A was not verified in the trial comparing B to A, because the active comparator served as the sole reference, small, cumulative slippage in efficacy may go undetected across drug generations. In therapeutic areas with many competing agents, this is a recognized regulatory concern (31, 32). In migraine prevention, where the CGRP class has produced multiple approved agents with similar but not identical efficacy profiles, the risk of bio-creep (33) is not theoretical. Addressing it requires that non-inferiority margins be anchored, at least in part, to historical placebo-controlled data from the reference active comparator's own pivotal program, not simply to the active comparator's point estimate from an earlier head-to-head trial.

## The problem of non-inferiority margin determination

A third challenge is the absence of consensus on what constitutes a clinically meaningful non-inferiority margin in migraine prevention. The minimal clinically important difference (MCID) for reduction in monthly migraine days has been estimated variably in the literature, with values of approximately 1.5–2.0 days per month commonly cited, though these estimates are themselves derived from studies with heterogeneous designs and patient populations. Non-inferiority margins that are too generous risk permitting drugs with meaningfully inferior efficacy to reach the market; margins that are too stringent make active-comparator trials practically infeasible, inadvertently reinforcing the default to placebo. Regulatory guidance on non-inferiority margins for migraine prevention—specifically distinguishing between episodic and chronic migraine and accounting for baseline frequency and prior treatment history- remains underdeveloped relative to guidance in other therapeutic areas (34–36). Developing such guidance, potentially through joint academic-regulatory working groups drawing on the now-substantial database of CGRP-era pivotal trial data, represents a methodological priority that would directly enable the transition away from placebo-controlled designs.

These methodological concerns are genuine, and the manuscript does not minimize them. The ethical implication, however, is not that the placebo must remain the permanent default while these challenges remain unresolved, but that the field bears a responsibility to resolve them. Assay sensitivity can be preserved through three-arm designs and robust interim monitoring; bio-creep can be managed by anchoring non-inferiority margins to placebo-controlled historical data; and MCID consensus can be developed from the rich trial database generated in the CGRP era. The persistence of placebo as the methodological default reflects, in part, a failure of investment in these design innovations, an inertia that is increasingly difficult to justify on either scientific or ethical grounds when effective, mechanism-specific preventives are available and when trial participants bear the cost of that inertia in months of preventable disability.

## The failure of beneficence and non-maleficence

Under beneficence and non-maleficence, the central ethical question is no longer abstract: what does “placebo” mean for a person with frequent migraine in 2026? To assign such a participant to a placebo for 12 to 24 weeks is not benign; it is months of predictable, preventable attacks, pain, lost function, and repeated reliance on acute medication. In acute migraine trials, placebo exposure is typically brief, and rescue is available within hours. In preventive trials, by contrast, a placebo can mean a whole season of untreated neurological disease (24–26).

This harm is not limited to transient discomfort. Recurrent attacks are themselves disabling, and the behavioral and pharmacological consequences of uncontrolled migraine (missed work, reduced activity, escalating acute-medication use) are predictable. Medication overuse and high baseline headache frequency are well-established risk factors for chronic headache and medication-overuse headache (MOH), and prospective data suggest that a subset of individuals with episodic headache progress to a chronic state within a year. While any single trial cannot be blamed for an individual's long-term trajectory, prolonged placebo exposure can plausibly increase risk by allowing sustained high-frequency disease and higher acute-medication exposure.

The ethical point does not require extreme estimates of rapid chronification. It is sufficient that investigators can foresee and prevent substantial suffering and downstream risk in the placebo arm, given that effective, migraine-specific preventives exist. In this context, prolonged placebo is increasingly difficult to defend as a necessary scientific inconvenience; it constitutes avoidable harm.

### Differential justification for placebo use according to patient characteristics

Placebo use is difficult to justify in patients with significant disability, those at risk of rapid chronification, those with existing medication overuse or risk factors for developing it, and those with high-frequency migraine, particularly when previous preventive therapy has already failed. Since risk factors for chronification and MOH are well documented, and high-frequency episodic migraine is itself a risk factor, only a narrow subgroup of patients may be suitable for placebo-controlled trials. Patients with low-to-moderate migraine frequency who have been screened for relevant comorbidities and risk factors, and who have not previously failed preventive therapy, may represent a subgroup in whom placebo is more justifiable—but this subgroup does not represent the majority of patients who typically enroll in prevention trials.

## Justice and the conversion of trials into access mechanisms

Justice concerns arise when trial participation becomes a substitute for access to standard care. In the United States, CGRP-targeted therapies are frequently subject to prior authorization and step-therapy requirements, with coverage limitations and denial patterns that vary across payers (11). In the United Kingdom, NICE technology appraisals may recommend CGRP agents only after multiple preventive failures, and access can be shaped by local commissioning, waiting lists, and service capacity (10). These constraints are not merely administrative: they structure who can realistically obtain CGRP therapy outside a trial.

When an effective, disease-specific preventive exists but is inaccessible to many, enrolment is often not pure altruism—it may be the only plausible route to care. In that setting, randomization to a placebo can function as a lottery for relief. The voluntariness of consent is strained not because patients cannot understand randomization, but because background injustice leaves them with few meaningful alternatives. Recent analyses of the U.S. migraine landscape have cautioned that placebo-controlled trials may disproportionately recruit individuals for whom trial participation is the only realistic path to advanced therapy, thereby amplifying inequities and distorting consent (6, 12).

This does not imply that all placebo-controlled migraine trials are exploitative. It does imply that, in the CGRP era, justice cannot be treated as an externality. Trial design choices interact with reimbursement systems, and the ethical burden of placebo grows when the very existence of placebo recruitment depends on structural barriers to care.

## Regulatory lag: why placebo remains the methodological default

One reason migraine trial ethics has not kept pace with therapeutic innovation is that regulatory guidance still frequently treats placebo-controlled designs as the evidentiary default. The U.S. Food and Drug Administration's 2023 draft guidance for migraine prevention recommends randomized, double-blind, placebo-controlled parallel-group trials as the standard approach for confirmatory efficacy evaluation, while acknowledging that active control may be appropriate in some circumstances (31). The European Medicines Agency guideline on migraine prophylaxis similarly emphasizes placebo-controlled methodology as the conventional standard for establishing efficacy (32).

This regulatory posture reflects legitimate scientific concerns, particularly assay sensitivity in a condition with variable outcomes and substantial placebo response. But it also creates inertia. When regulators expect a placebo, sponsors design one; and when sponsors design a placebo, ethics committees and investigators are asked to treat prolonged placebo exposure as routine. The result is a mismatch between professional standards of clinical care (8), payer-mediated access (10–12), and the ethical defaults embedded in trial guidance. Ethics-focused critiques in the headache literature have argued that routinized placebo use can drift into “placebo following placebo” designs with questionable scientific or ethical value (34).

### The CGRP era: a field that has become a victim of its own progress

**Figure 1:**
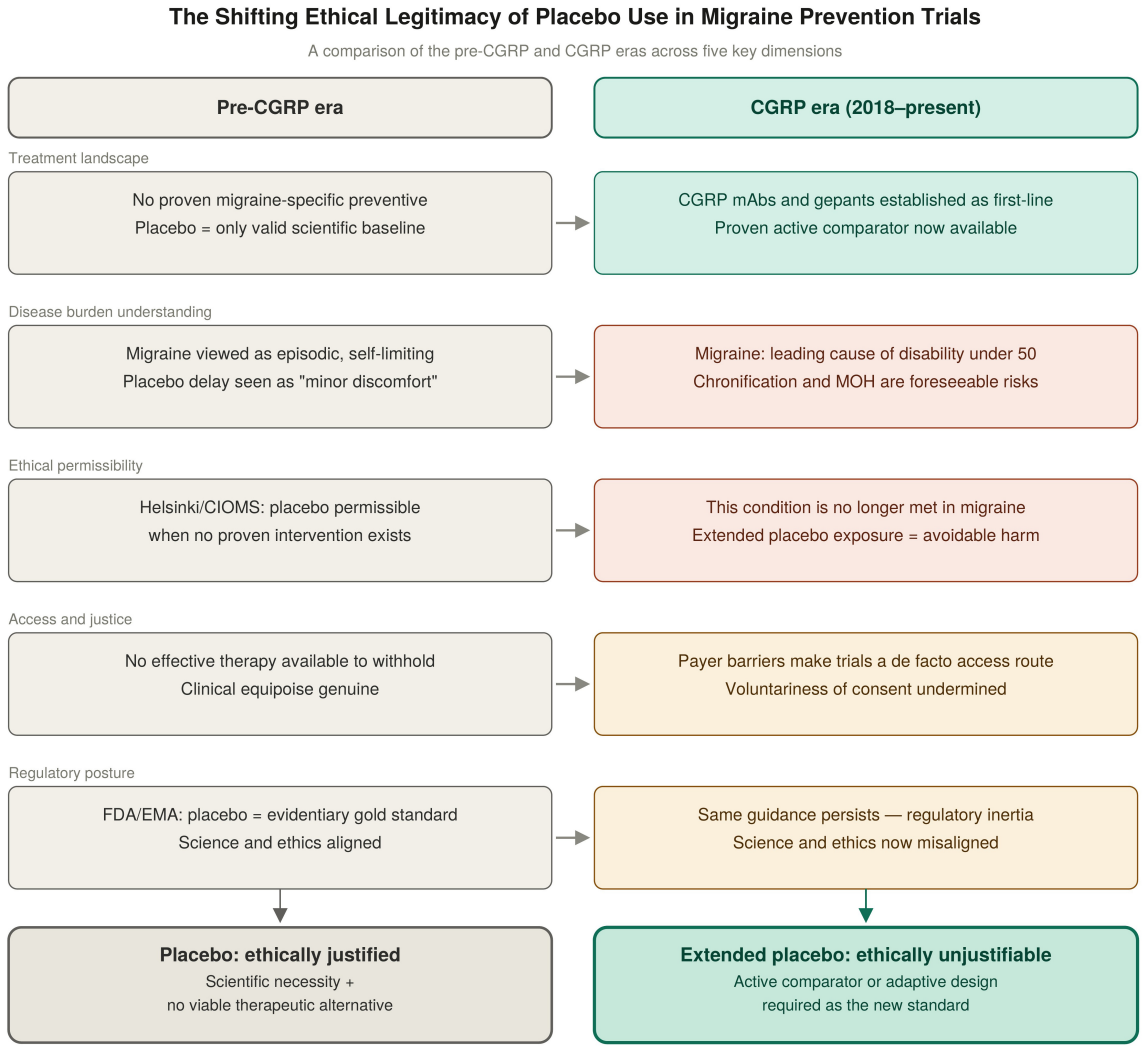
The shifting ethical legitimacy of placebo use in migraine prevention trials. Five dimensions of the pre-CGRP and CGRP eras are compared. Row color indicates the category of change: gray, pre-CGRP baseline; teal, therapeutic advance; coral, increased ethical burden; amber, tension and regulatory lag; AHS, American headache society; CGRP, calcitonin gene-related peptide; DALY, disability-adjusted life year; GBD, global burden of disease; MOH, medication-overuse headache.

The introduction of CGRP-targeting therapies represents a watershed moment in headache medicine. These agents are the first preventives specifically engineered for migraine pathophysiology. Their high efficacy and favorable tolerability profiles have effectively ended the era of “no proven treatment” (8, 9) (Figure 1).

When a field possesses highly effective, disease-specific treatments, the bar for clinical equipoise is raised. We are no longer searching for a light in the dark; we are comparing different shades of light. The CGRP era does not imply that placebo controls are never permissible in migraine research. It implies that prolonged placebo exposure should no longer be the default in preventive drug trials when highly effective, migraine-specific therapies exist. Scientific rigor and ethical responsibility are not competing aims; they must now be aligned by design.

## Proposed ethical alternatives for the modern era

To modernize migraine research, we must adopt designs that maintain scientific rigor while ensuring all participants receive a therapeutic floor of care.

### Active-comparator or non-inferiority designs as the default

Where a proven preventive intervention exists, new agents should generally be evaluated against best-available active therapy, or within non-inferiority frameworks that preserve assay sensitivity through pre-specified margins and robust trial conduct (13, 15, 16, 25, 30).

### Adaptive and rescue frameworks that minimize placebo duration

If a placebo is scientifically necessary to establish efficacy, it should be time-limited and paired with early-escape criteria, rescue options, and interim monitoring to curtail exposure without compromising interpretability. Adaptive trials use pre-specified rules to adjust the trial based on accumulating interim data. For instance, if early data show that one patient group is responding poorly or that a specific dose is ineffective, researchers can adjust dosing, drop ineffective arms, or stop enrolling patients into less effective groups, thereby minimizing the total patient-time spent on suboptimal therapy.

### Add-on designs

In an add-on design, the investigational drug is tested against a placebo added to the patient's existing stable preventive therapy. This ensures that even participants in the control group continue to receive a baseline of proven care, while still allowing measurement of the incremental efficacy of the new intervention. This approach is particularly relevant for difficult-to-treat populations.

### Crossover designs

In a crossover design, no participant is assigned exclusively to a placebo for the full trial duration. Placebo exposure is brief, and each participant serves as their own control, making the placebo phase more ethically justifiable. The low dropout rates observed in crossover trials in migraine patients provide evidence of the ethical superiority of this approach.

### Shortened placebo periods with early-escape criteria

If regulatory bodies require a placebo arm to establish a clean efficacy signal, its duration should be minimized. Protocols must include pre-specified early-escape criteria: if a participant's attack frequency exceeds a defined threshold or their disability reaches a specified level (as measured by the MIDAS or HIT-6 scales), they are immediately transitioned to active therapy. This provision protects participants from prolonged suffering without compromising scientific validity.

### Mandatory post-trial access to effective therapy

When participation in research substitutes for access to care, justice requires concrete post-trial access commitments for participants who benefited or who remained in need of effective prevention (5, 6).

Methodological innovation can complement, rather than replace, these ethical safeguards. For example, open-label placebo approaches are being explored in migraine and may offer ethically acceptable ways to harness contextual effects without deception. Pragmatic trials embedded in routine care and real-world evidence studies can answer comparative and implementation questions without long placebo exposure. External or historical controls may also be considered in limited circumstances but require careful attention to bias and confounding (13).

## Discussion

The transition away from PCTs in migraine prevention is not just a scientific evolution; it is a moral one. The gold standard of the 1990s has become the ethical compromise of the 2020s. While PCTs were once a necessary tool for a burgeoning field, the modern era of neurology has outgrown them (Figure 1).

The pharmaceutical industry, regulatory agencies (FDA/EMA), and academic societies must collaborate to define new benchmarks for drug approval. This includes accepting non-inferiority margins as evidence of clinically meaningful efficacy and prioritizing head-to-head data to support clinical decision-making. The goal of migraine research should not merely be to prove a drug is better than nothing, but to demonstrate it has a meaningful place within an established landscape of care.

## Conclusion

CGRP-targeting therapies and gepants have moved migraine prevention into a new therapeutic era, one in which prevention is no longer limited to symptom management but is increasingly mechanism-based and biologically targeted. Professional societies now position CGRP-targeting therapies as first-line options, signaling an updated standard of care (8). Against that backdrop, prolonged placebo-controlled prevention trials represent a widening mismatch between clinical reality and research ethics.

Ethical guidance permits a placebo only under specific conditions—most importantly, when no proven effective intervention exists or when a placebo is methodologically necessary without exposing participants to serious or irreversible harm (5, 6, 14). In migraine prevention, extended placebo exposure predictably entails weeks to months of uncontrolled attacks and disability and can encourage acute-medication escalation that increases risk for MOH and chronification (18, 19, 26). We contend that migraine prevention no longer meets the “negligible risk” threshold: months of untreated attacks are predictably associated with substantial disability, increased acute-medication exposure, and elevated risk of MOH and chronification. Justice concerns amplify these risks because payer restrictions and prior authorization make trial participation a practical route to otherwise inaccessible treatment for many patients, potentially distorting voluntariness and informed consent (10–12). We propose an updated ethical standard for migraine prevention trials. The path forward lies in adaptive designs and active-controlled trials that respect both the science we have built and the patients we serve. Migraine provides a paradigmatic case in neuroethics in which scientific rigor must evolve in tandem with rapidly changing standards of care (20).

For a neuroethics audience, migraine trials offer a concrete test case: as therapeutics become more effective and disease-specific, trial ethics must shift from permissive historical defaults toward designs that respect contemporary standards of care and structural inequities in access. A new standard-of-care mandate in migraine research should make active-comparator or non-inferiority designs the default, constrain placebo exposure through adaptive and rescue frameworks, and require enforceable post-trial access to effective agents.
